# Effects of Between-Sprint Running Intensity on Repeated-Sprint Performance in Professional Soccer Players

**DOI:** 10.3390/sports14030097

**Published:** 2026-03-03

**Authors:** Gregory Bizas, Ilias Smilios, Pierros Thomakos, Gregory C. Bogdanis

**Affiliations:** 1 Department of Physical Education & Sport Science, Democritus University of Thrace, 69132 Komotini, Greece; gregorybizas@hotmail.com (G.B.); ismilios@phyed.duth.gr (I.S.); 2 School of P.E. and Sport Science, National and Kapodistrian University of Athens, 17237 Athens, Greece; pthom@phed.uoa.gr

**Keywords:** repeated-sprint ability, blood lactate concentration, active recovery, fatigue

## Abstract

This study examined the effects of two different intensities of running between repeated sprints and compared them with passive recovery. Thirteen professional soccer players performed two sets of six 30 m sprints on three randomly assigned occasions. A 5 min passive rest period separated the two sets, while sprints were interspersed with either passive standing, running at 95% of the first lactate threshold (MOD) and running at maximum aerobic speed (HIGH). Performance decrements were greater in HIGH than MOD at the last sprint in both sets (set 1: 5.8 ± 4.2% vs. 2.6 ± 3.2%, *p* = 0.07; set 2: 9.1 ± 4.5% vs. 4.0 ± 6.1%, *p* = 0.016). Acceleration (0–15 m) was more affected than maximal-speed running (15–30 m) (condition × sprint interaction: *p* < 0.001). Mean and peak heart rate were higher in both running conditions than passive (*p* < 0.05), with no difference between MOD and HIGH. Blood lactate showed a significant set × condition interaction (*p* < 0.001), peaking at 13.6 ± 2.7 mmol·L^−1^ in HIGH, while blood lactate responses to passive and MOD were similar and peaked after the second set of sprints (10.7 ± 2.1 and 11.5 ± 2.8 mmol·L^−1^, respectively). Between-sprint running intensity markedly influenced fatigue development during repeated-sprint exercise. The HIGH condition elicited greater metabolic strain and performance decrements than MOD or passive conditions. Within the present protocol, passive standing was associated with smaller decrements in repeated-sprint performance despite high heart rate and blood lactate responses.

## 1. Introduction

Soccer is a team sport characterized by an intermittent activity pattern in which brief high-intensity exercise bouts, such as high-speed running and sprinting, are interspersed with lower intensity activities, such as walking and jogging [1,2]. Even though players run a total distance of around 10–12 km, high-speed running performance (i.e., distance run at speeds > 19.8 km·h^−1^), which constitutes only 7–9% of the total distance [2], is important for a successful result [3,4,5]. Several studies have shown that the frequency and intensity of high-speed running and sprinting are positively associated with match outcome [4,6,7,8] and are indicative of the players’ fitness level [9]. The ability of the player to cover large total distances with high-speed running depends not only on anaerobic power, but also on the ability to recover between high-intensity bouts, which enables repetition of fast runs during the match [10,11]. This capacity to repeatedly perform short, high-intensity sprints with limited recovery is commonly referred to as repeated-sprint ability (RSA). RSA has been widely used both as a performance assessment and as a training methodology in team sports to evaluate fatigue resistance and recovery between successive sprint efforts [12,13]. At high levels of performance, players cover on average between 750 and 900 m at speeds faster than 19.8 km·h^−1^ (high-speed running) and 200–230 m at speeds faster than 25.2 km·h^−1^ (sprinting) [6]. The number of sprints during a match is between 8 and 12 [14], while some of those sprints may occur in short succession, separated by very short recovery intervals of 2–65 s [2,15].

The recovery time between very high-intensity running bouts during a soccer match is on average 60–80 s [11,14], and it is known that this time is not sufficient for full recovery of muscle metabolism and maintenance of sprint performance [16,17]. Short recovery times between high-intensity activities are also seen in “worst-case scenarios” during games, which may be decisive for the final score [18]. Previous studies using repeated sprints as a model to study fatigue showed that performance over a test of 15 × 40 m sprints was maintained only when the recovery between bouts was 120 s, while fatigue was evident when recovery was reduced to 30 or 60 s [19]. Most studies examining changes in performance during repeated sprints in soccer players used passive recovery, which typically lasted between 10 and 30 s, aiming to induce measurable fatigue [20]. In the few studies where active recovery was used, the intensity was very low and uncontrolled [16,21]. Importantly, although several studies have included active recovery between repeated high-intensity efforts, its intensity has rarely been prescribed or manipulated according to clearly defined physiological reference points. In the present study, we focus on the intensity of locomotor activity performed between sprint efforts (hereafter termed between-sprint running intensity). In particular, running between sprint efforts at very high intensities, such as maximal aerobic speed (MAS), has not been systematically examined within a controlled repeated-sprint framework using individualized physiological anchors. During match play, recovery between high-intensity efforts may range from walking to high-intensity running [21], while during the most intense periods of the match the duration of recovery intervals may be as short as 20–40 s. As a result, higher between-sprint running intensities may decrease performance, as it is detrimental for phosphocreatine resynthesis, muscle lactate clearance and muscle oxygenation [22,23,24,25]. Even at a low intensity (slow running at 7.2 km·h^−1^), studies show greater reductions in sprint performance compared to passive recovery [22]. Active recovery at an intensity below the lactate threshold would theoretically allow for the elimination of metabolic byproducts and minimal activation of glycolysis with lactate formation, thereby causing minimal disruption to sprint performance. However, it is worth noting that during intense periods of soccer matches, players perform high-intensity runs at intensities faster than that corresponding to lactate threshold or even VO_2_max during the recovery periods between repeated sprints [26,27,28]. An intense five-minute period in soccer may involve several sprint actions interspersed with sustained high-intensity running, particularly during transitional phases where a team attacks, loses possession, retreats quickly to defend, applies immediate pressure on the opponent, and then initiates a subsequent attacking phase [26,27,28]. This may lead to incomplete recovery following a sprint action due to the demanding nature of high-intensity running and the progressive deterioration of sprint-running performance.

To examine responses across a broader range of physiological demands, the present study manipulated the intensity of running performed between sprint efforts using individualized anchors, including a condition at maximal aerobic speed. Incorporating this high-intensity condition allows investigation of fatigue responses under elevated between-sprint locomotor load within a controlled experimental framework, extending previous work that has primarily examined passive or low-intensity conditions. Nevertheless, despite evidence that players may be required to perform sustained high-intensity running between successive sprint actions during the most demanding phases of match play, no study has systematically examined the effects of manipulating between-sprint running intensity at very high levels—approaching maximal aerobic speed—on repeated-sprint performance.

Repeated-sprint tests are used to examine performance responses under controlled manipulation of sprint distance, interval duration, and running intensity between efforts [29,30]. Accordingly, the present study focuses on sprint distance, interval duration, and running intensity between efforts as experimentally controlled variables.

However, the typical repeated-sprint tests include short sprints (5–7 bouts of ~4–5 s), which are repeated after a short passive standing interval (~20–30 s) [30,31]. Although some studies have reported associations between performance in a 5 × 30 m sprint protocol and match sprinting performance [32], the extent to which such tests reflect match demands remains debated [21]. Given the limited evidence regarding the role of different between-sprint running intensities, we implemented a repeated-sprint protocol consisting of two standardized high-intensity blocks separated by 5 min of rest. This approach allowed between-sprint running intensity to be precisely prescribed and controlled, while maintaining a high level of experimental standardization. Accordingly, prescribing running at intensities approaching maximal aerobic speed was used to impose a controlled and individualized high locomotor load between sprint efforts, facilitating examination of repeated-sprint responses. The present study therefore adopts a reductionist approach to isolate the influence of between-sprint running intensity under controlled conditions rather than attempting to reproduce the full complexity of match play.

The aim of the present study was to examine how between-sprint running intensity across a wide physiological range—from passive conditions, to sub-lactate-threshold running, to very high-intensity running at maximal aerobic speed (MAS)—affects repeated-sprint performance. We hypothesized that repeated-sprint performance would be lower during running between sprints compared with a passive condition, and that the higher between-sprint intensity would be more detrimental to sprint performance.

## 2. Materials and Methods

### 2.1. Participants

Power analysis using the repeated-measures, within-subject main effect of condition in the planned 3-way repeated-measures analysis of variance (G-Power software, v. 3.1.9.2, Universität Kiel, Kiel, Germany) indicated a minimum of 12 participants completing all conditions, based on a power of 0.80, alpha of 0.05, correlation coefficient of 0.5 between repeated measures, and effect size d = 0.80. This was based on previous studies, where the effect size for sprint performance was between medium and large (Cohen’s d = 0.80 and 1.20) [19,25].

Thirteen professional soccer players (from divisions 1 and 2 of the Greek Super League) volunteered to participate in the study (age: 22.5 ± 1.8 years, height: 181.0 ± 6.6 cm, body mass: 75.9 ± 6.2 kg, body fat percentage: 8.2 ± 3.1%). The positions of the players were: 4 central defenders, 5 wingers and full backs, 2 forwards, and 2 central midfielders. All players had no musculoskeletal injuries for at least 6 months before the study and were not taking any nutritional supplements or medication. The study protocol and all measurements were thoroughly explained to the players, who then signed an informed consent. Players were also informed about their right to stop their participation any time without providing any explanation for their decision. The study protocol and all procedures were in accordance with the Code of Ethics of the World Medical Association (Helsinki Declaration of 1964, as revised in 2024), and was approved by the local Institutional Review Board (1495/15-3-2023).

### 2.2. Experimental Design

A randomized crossover repeated-measures design was employed to compare changes in sprint performance, blood lactate and heart rate during three conditions: (a) passive standing between sprints, (b) running between sprints at moderate intensity (MOD) corresponding to 95% of the first lactate threshold speed, and (c) running between sprints at high intensity corresponding to the maximum aerobic speed (HIGH), as determined by the Yo-Yo Intermittent Recovery Level 1 Test (Yo-Yo IRT Level 1). These conditions were implemented as different between-sprint running intensities rather than recovery strategies. The participants were recruited immediately after the end of their leagues and performed light individual training during their participation in the study. All measurements were made at the facilities of the training center of the national soccer teams.

Running between sprints at a moderate intensity (95% of the lactate threshold speed) was selected because it would allow metabolic byproduct elimination from the sprinting actions while minimizing the activation of glycolysis with lactate formation. A high intensity (maximal aerobic speed) was selected because during a soccer match, players may perform high-intensity runs (>14.4 km·h^−1^) between repeated sprints when, for example, returning to defense after an attacking action or when trying to cover space to control movements of opposition players. Prescribing running at MAS therefore allowed examination of responses under a controlled high inter-sprint physiological load, serving as a standardized boundary condition.

### 2.3. Procedures

#### 2.3.1. Anthropometrics and Maximum Speed Measurement

The participants were familiarized with the procedure of the test during two preliminary visits one or two days apart. In the first main session, basic anthropometry and body composition measurements were taken and were used for descriptive purposes only. Height and weight of the players were measured with a digital balance with an attached stadiometer (Seca 769 digital scale with Seca 220 stadiometer, Seca GMBH, Hamburg, Germany). Body density was estimated by measuring skinfold thickness at the chest, triceps, subscapular, abdominal, thigh, mid-axillary and suprailiac sites, using a 7-site equation [33]. The percentage of body fat was obtained from the Siri equation.

Then, following a standardized warm-up that included 6 min low-intensity running over a 20 m course combined with skipping of different types, heel kicks, dynamic stretching, light single- and double-leg jumping, short accelerations, and one 10 m sprint. Sprint performance over three 40 m sprints separated by 5 min of rest was determined. Split times were obtained for 10, 30 and 40 m using photocells (Kit Racetime2 Light Radio, Microgate, Italy). Maximum running speed was calculated from the 30 m and 40 m times of the best sprint, using the following formula:

Maximum running speed (km·h^−1^) = ((10 m/(time 40 m − time 30 m)) × 3.6)

In all athletes the maximum speed was achieved either in the first or the second attempt.

#### 2.3.2. Determination of the Blood Lactate Threshold

After 2–3 days from the first preliminary session, the players performed a submaximal outdoor shuttle running test over a distance of 100 m with progressively increasing speed to determine lactate threshold from the relationship between blood lactate, running speed and heart rate (Polar H9 heart rate monitor, Polar Electro Oy, Kempele, Finland). The test started at a running speed of 8 km·h^−1^, which increased by 2 km·h^−1^ every 3 min until reaching 16 km·h^−1^. Control of running speed was determined by an audio signal played through computer speakers using a Finis Tempo Trainer Pro device (Tempo Trainer Pro; FINIS, Inc., Tracy, CA, USA) to generate the sound accurately. After each 3 min stage, participants briefly stopped for 30 s to provide a capillary blood sample, which was analyzed for lactate concentration using the Lactate Scout+ analyzer (EKF Diagnostics, Cardiff, UK). The individual relationship between lactate concentrations and running velocities was determined using a nonlinear regression model: y = b × exp(x/c) + a, where y = lactate concentration, x = running speed and a, b and c are constants. The first lactate threshold (LT1) was defined as the running speed corresponding to an increase in blood lactate concentration of 0.5 mmol·L^−1^ above baseline, where baseline was calculated as the mean lactate concentration across the first 2–3 stages of the incremental test [34,35].

#### 2.3.3. Yo-Yo Intermittent Recovery Test Level 1

Two to four days after the submaximal test, the athletes returned to the training complex and performed the Yo-Yo Intermittent Recovery Test Level 1 (Yo-Yo IRT Level 1) to exhaustion, to determine the maximum aerobic speed. The test consisted of repeated 2 × 20 m runs back and forth with a progressive increase in running speed (Bangsbosport.com). Between each running bout there was a 10 s active rest period, consisting of 2 × 5 m of light jogging. When the participants failed twice to reach the finishing lines, the distance covered was recorded and kept as the test result. The VO_2_max was estimated for Yo-Yo IR1 test from the following equation [9]:

VO_2_max (mL^−1^·kg^−1^·min^−1^) = IR1 distance (m) × 0.0084 + 36.4

The Yo-Yo Intermittent Recovery Test Level 1 provides an estimate of maximal aerobic capacity and maximal running speed under intermittent conditions; thus, the maximal speed attained during the test is used as a practical surrogate of maximal aerobic speed for prescribing running intensities in intermittent exercise settings [9]. Using MAS and LT1 as individualized anchors ensured comparable relative physiological stress across participants despite differences in absolute running speed.

#### 2.3.4. Repeated Sprints Trials

The athletes participated in three experimental conditions performed in a randomized and counterbalanced order, separated by 4–7 days of recovery. All conditions involved two sets of six 30 m sprints, interspersed with 60 s of recovery and 5 min rest between the two sets. On one occasion, the 60 s of recovery between sprints was passive, while on the other two occasions the middle 30 s of the 60 s recovery periods involved running between sprints, i.e., 30 s of running either at a speed corresponding to 95% of the individual speed at the lactate threshold (moderate between-sprint intensity: MOD) or at a speed corresponding to the speed attained at the end of the Yo-Yo IR1 test (high between-sprint intensity: HIGH). The distance that the subjects had to cover was determined by their individual running speed at L1 and MAS. During the 30 s running period between sprints, players had to cover 86 ± 6 m in the MOD and 139 ± 3 m in the HIGH condition. This distance was covered over a straight course of 43 ± 3 m and 70 ± 2 m, respectively (there and back), while a steady pace was maintained with the aid of auditory signals emitted by a Finis Tempo Trainer Pro device (Tempo Trainer Pro; FINIS, Inc., Tracy, CA, USA) via a beep sound every 7.5 s in the MOD and 5.0 s in the HIGH condition (approximately every 20–25 m). Sprint performance time was measured for the first 15 m, the last 15 m and the total 30 m of each sprint using photocells (Kit Racetime2 Light Radio, Microgate, Italy).

**Figure 1:**
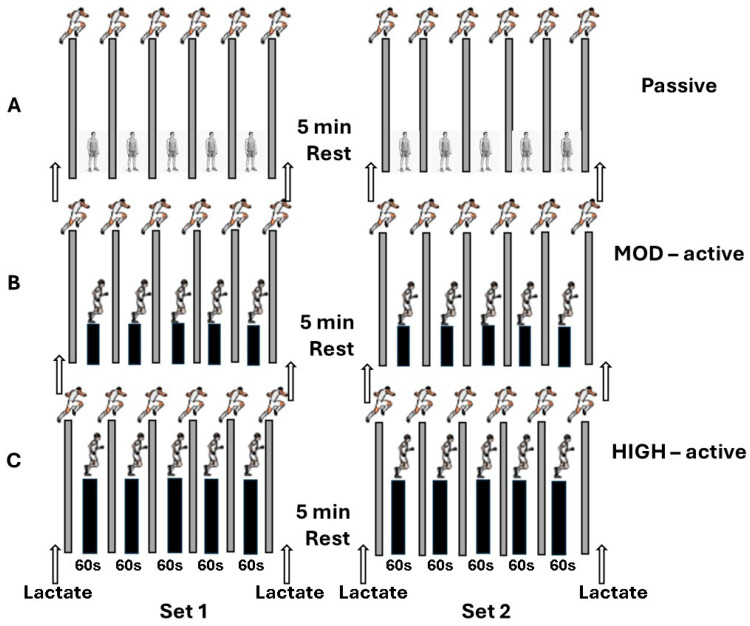
Schematic representation of the study protocol. The three conditions are (**A**) passive standing between sprints, (**B**) running between sprints at an intensity corresponding to 95% of lactate threshold speed (MOD), and (**C**) running between sprints at an intensity corresponding to maximal aerobic speed (HIGH). Blood lactate samples were taken before and after each sprint set; gray bars show 30 m linear sprints; black bars show running performed during the middle 30 s of the 60 s interval between sprints.

Blood lactate concentration was measured in fingertip capillary samples collected before the start of the first sprint, immediately after completion of the first set, prior to the second set, and after the end of the second set. The experimental design is illustrated in Figure 1.

### 2.4. Statistical Analysis

Data are presented as mean ± standard deviation (SD). Statistical analyses were performed using SPSS software (Version 29, IBM, Chicago, IL, USA). A three-way analysis of variance (ANOVA) with repeated measures on all factors was applied, including three between-sprint intensity conditions (passive, MOD, and HIGH), two sets, and six repetitions of 30 m sprints (3 × 2 × 6 within-subject design). Performance times during the repeated-sprint protocols were analyzed for the entire 30 m as well as separately for two segments (0–15 m and 15–30 m). Blood lactate responses were analyzed using two-way ANOVA (condition × time), while average and peak heart rate responses were analyzed with repeated-measures two-way ANOVA (condition × set). When a significant interaction or main effect was detected, Tukey’s post hoc test was conducted. Effect size in the ANOVAs was determined using partial eta squared (η^2^), which was classified as small (>0.01–0.059), medium (>0.06–0.139) and large (>0.14). Assumptions of repeated-measures ANOVA were assessed prior to analysis. Normality of residuals was evaluated using the Shapiro–Wilk test, which is appropriate for small sample sizes, and sphericity was assessed using Mauchly’s test. When violations of sphericity were detected, Greenhouse–Geisser corrections were applied. To examine the relationship between aerobic fitness and fatigue, Pearson correlation coefficients were calculated between MAS, LT1, and the decline in sprint performance during the repeated-sprint test. Statistical significance was set at *p* < 0.05.

## 3. Results

### 3.1. Preliminary Tests Results

Table 1 shows the results of the preliminary tests, i.e., the Yo-Yo IR1 test for estimating maximum aerobic speed, the 40 m sprint test for determining maximum sprinting speed, and the submaximal running test for determining LT1.

### 3.2. Repeated Sprints Performance

**Figure 2:**
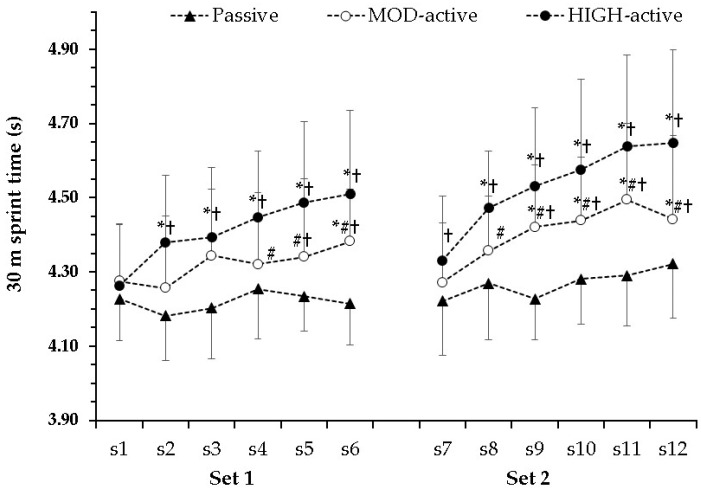
Repeated-sprint performance (30 m sprint time) during the three conditions. Values are mean ± SD. MOD: running between sprints at 95% of first lactate threshold speed (LT1); HIGH: running between sprints at maximal aerobic speed (MAS); *: *p* < 0.01 from sprint 1 of the same condition; #: *p* < 0.01 from the corresponding sprint of the HIGH condition; †: *p* < 0.01 from the corresponding sprint of the passive condition.

The three-way ANOVA for 30 m sprint performance showed a significant condition × sprint interaction (*p* < 0.001, η^2^ = 0.34—large), but non-significant condition × set × sprint and condition × set interactions (*p* = 0.15, η^2^ = 0.11—medium and *p* = 0.12, η^2^ = 0.16—large, respectively). Post hoc tests for the condition × sprint interaction showed that there was no change in sprint performance in the passive standing condition (Figure 2). In contrast, sprint performance started to decline from the sixth sprint onwards in the MOD condition and as early as from the second sprint in the HIGH condition, indicating a strong influence of between-sprint running intensity on fatigue development during repeated sprints. The difference between corresponding sprints performed in the passive standing condition and the MOD condition was evident from sprint 5 onwards, while the difference between the MOD and the HIGH conditions was seen from the fourth sprint. It is noteworthy that 5 min of rest was adequate for recovery of sprint performance, albeit only in the first sprint of the second set. The percentage of reduction in sprint performance was twice as high in the HIGH compared with the MOD condition at the end of set 2 (9.1 ± 4.5% vs. 4.0 ± 6.1%, *p* = 0.016, d = 0.95, large), whereas in set 1 this difference did not reach significance (5.8 ± 4.2% vs. 2.6 ± 3.2%, *p* = 0.07, d = 0.86, large).

The results for sprint performance time separated into two parts (0–15 and 15–30 m) are presented in Table 2. The three-way ANOVA for performance time during the first 15 m of each 30 m sprint (0–15 m) showed a significant condition × sprint interaction (*p* < 0.001, η^2^ = 0.23), but non-significant condition × set × sprint and condition × set interactions (*p* = 0.11, η^2^ = 0.12 and *p* = 0.33, η^2^ = 0.08, respectively). Similarly, the three-way ANOVA for performance time during the last 15 m of each 30 m sprint (15–30 m) showed the same pattern, i.e., a significant condition × sprint interaction (*p* < 0.001, η^2^ = 0.31), but non-significant condition × set × sprint and condition × set interactions (*p* = 0.64, η^2^ = 0.06 and *p* = 0.12, η^2^ = 0.16, respectively). Post hoc tests revealed that the first part of the sprint, i.e., 0–15 m, was more affected by between-sprint running intensity, especially in the second set of sprints, since performance times for the 0–15 m section were only affected by MOD, while performance in the 15–30 s section was similar to that in passive standing (see Table 2).

No statistically significant correlations were found between the speed at LT1, expressed either as an absolute value or as a percentage of MAS, and the drop in sprint performance during the repeated-sprint test in all conditions (r = −0.20 to 0.38, *p* > 0.05). Similarly, low and non-significant correlations were found between MAS and the drop in sprint performance during the repeated-sprint test (r = −0.18 to −0.54, *p* > 0.05).

### 3.3. Heart Rate Responses

Mean and peak heart rate during the two sets of sprints in each condition are presented in Table 3. The two-way ANOVA for both mean and peak heart rate showed a non-significant set × condition interaction (*p* = 0.102, η^2^ = 0.17 and *p* = 0.059, η^2^ = 0.21, respectively), with post hoc tests revealing an increase in HR from set 1 to set 2 in all conditions, and a significantly higher average and peak HR in MOD and HIGH from passive, with no difference between the two running conditions (*p* = 0.40 for mean HR and *p* = 0.06 for peak HR; see Table 3).

### 3.4. Blood Lactate Responses

**Figure 3:**
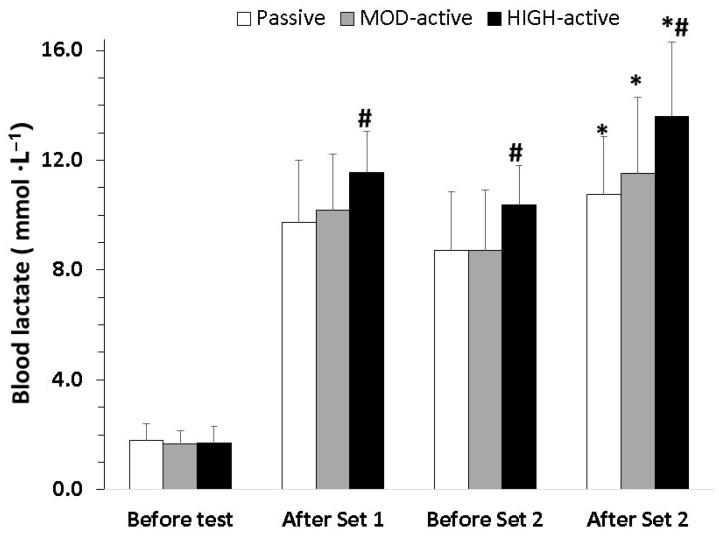
Blood lactate responses during the three conditions at four time points (before the test, after set 1, before set 2 and after set 2). Values are mean ± SD. MOD: running between sprints at 95% of lactate threshold; HIGH: running between sprints at maximum aerobic speed (MAS); *: *p* < 0.05 vs. after set 1 at the corresponding condition; #: *p* < 0.05 vs. passive and MOD at the corresponding time point.

Figure 3 shows the blood lactate responses during the three experimental conditions. The two-way ANOVA showed a significant set × condition interaction (*p* < 0.001, η^2^ = 0.27). The post hoc tests indicated that blood lactate increased equally in passive and MOD conditions after the first set of sprints (to 9.7 ± 2.3 and 10.2 ± 2.1 mmol·L^−1^, respectively; see Figure 3) and peaked after the second set of sprints (10.7 ± 2.1 and 11.5 ± 2.8 mmol·L^−1^, respectively; see Figure 3). However, blood lactate concentration was higher in HIGH than the other two conditions at all time points during exercise, peaking at 13.6 ± 2.7 mmol·L^−1^ immediately after the second set of sprints (Figure 3).

## 4. Discussion

The main finding of the present study was that between-sprint running intensity markedly influenced repeated-sprint performance, with progressively greater fatigue observed as the intensity of running performed between sprint efforts increased. Moderate-intensity running performed below the lactate threshold resulted in greater performance decrements compared with passive standing, while running performed at maximal aerobic speed led to the most pronounced impairment in sprint performance. These findings demonstrate that exercise performed between sprint efforts is not metabolically neutral [36] and that the intensity of locomotor activity between sprints is a key determinant of fatigue development during repeated sprinting.

To examine this effect under controlled conditions, the protocol incorporated two physiologically anchored between-sprint running intensities—95% of lactate threshold speed and maximal aerobic speed—and compared them with passive standing. Accordingly, the protocol should be interpreted as examining responses to manipulated between-sprint running intensity under controlled conditions, rather than as replicating the complexity of match scenarios.

Running below the lactate threshold between sprints has been suggested to promote recovery of power output [17], although this is not always the case [16,37]. As shown in Figure 3, blood lactate responses were similar in the MOD and passive standing conditions, indicating that low-intensity running between sprints (corresponding to 65.0 ± 5.5% of MAS) did not exert either a positive or negative effect on blood lactate concentration. However, heart rate was substantially higher in the MOD condition (e.g., 82.1 ± 3.0% vs. 74.0 ± 4.0%; see Table 3), indicating higher metabolic demand and potentially reduced phosphocreatine (PCr) resynthesis, which would negatively affect performance [38,39]. In line with this, a significant decline in sprint performance was observed in the MOD condition compared with the passive condition, in which performance was maintained (Figure 2). These findings are consistent with previous studies comparing passive and active recovery, which have demonstrated that passive recovery (i.e., standing or walking) allows greater restoration of performance between sprints and reduces fatigue [8,17,22,24,25]. One possible explanation is that running between sprints requires substantial mitochondrial energy supply, which may not be sufficient to simultaneously support aerobic exercise and PCr resynthesis, unlike passive standing [24]. Spencer et al. [25], using a 6 × 4 s cycling protocol with passive and active recovery, showed that active recovery resulted in significantly higher muscle lactate levels and a strong tendency toward lower PCr content, based on muscle biopsies obtained from the vastus lateralis. These findings further support the notion that running between sprints at speeds above LT may impair PCr resynthesis and subsequent repeated-sprint performance. Our findings align with this previous work demonstrating greater fatigue during active compared with passive conditions and extend the literature by examining responses across a wider range of individualized between-sprint intensities.

When the intensity of running between sprints was increased to MAS, fatigue was more pronounced, reflecting the substantially greater metabolic demands imposed by elevated between-sprint locomotor load. Accordingly, the MAS condition should be interpreted as a controlled high inter-sprint physiological stress rather than a recovery strategy. The greater fatigue observed under this condition is consistent with the expected limitations in phosphocreatine resynthesis and increased metabolic strain when high-intensity activity is maintained between sprint efforts. These responses indicate that increasing between-sprint running intensity accelerates fatigue development during repeated sprinting. Previous studies have reported similar physiological and performance responses when a comparable number of repeated sprints (e.g., 15 × 6 s sprints) were performed with 1 min of passive recovery [36]. Other studies have demonstrated that sprint duration, recovery duration, and the exercise-to-rest ratio are key determinants of fatigue during repeated sprinting [31,40]. All these studies clearly showed that increasing sprint distance [31] and/or rest duration [40] can result in large differences in fatigue, with a 7.8% decline in sprint performance observed after 40 × 15 m sprints with a 1:6 work-to-rest ratio. However, the present study is the first to directly compare passive recovery of match-relevant duration with two individualized active-recovery intensities anchored to robust physiological landmarks (i.e., first lactate threshold and MAS). The factors determining the fatigue profile of skeletal muscle during intense exercise include muscle fiber composition, neuromuscular characteristics, high-energy metabolite stores, buffering capacity, ionic regulation, capillarization, and mitochondrial density [10]. Despite this, we did not observe correlations between sprint performance, fatigue indices, and measures of aerobic fitness such as MAS or lactate threshold. This may be attributable to the relatively narrow range of values observed for key parameters (e.g., MAS: 16.0–17.4 km·h^−1^; LT1: 9.5–12.9 km·h^−1^) and the relatively low sample size, which may limit the power of correlation analyses.

Fatigue during a match, particularly during crucial phases requiring repeated high-intensity actions, is a reminder of the role of fatigue in match success. Therefore, preparing players to perform during these intense periods is of clear practical importance. The MOD and HIGH protocols used in this study, with sprints separated by 1 min, may serve not only as controlled tools for assessing tolerance to increasing between-sprint running intensity. Notably, analysis of performance during the first and last 15 m of each sprint revealed that, in the second set of the MOD condition, fatigue was evident only in the first 15 m—that is, during the acceleration phase. Acceleration relies heavily on high ATP turnover rates and adequate PCr availability [10,41]. Accordingly, acceleration capacity is expected to be more adversely affected under conditions of greater metabolic fatigue. This effect may also extend to alterations in the contribution of different leg muscles during fatigued sprinting (e.g., gluteus maximus and hamstrings), which were not examined in the present study [42,43].

The current study has some limitations. The sample consisted of soccer players from different clubs with varying annual training programs, training loads, and competitive match exposure during the in-season period. Also, it should be considered that in the present study the between-sprint running intensity in the MOD condition was based on LT1, defined as a +0.5 mmol·L^−1^ increase above baseline; alternative LT1 definitions may yield slightly different running velocities and, consequently, influence between-sprint running intensity prescription and performance outcomes. Furthermore, playing position is an important determinant of the frequency, duration, and recovery characteristics of high-intensity efforts during a match [21]. Due to the limited sample size (n = 13) and positional heterogeneity, no conclusions could be drawn regarding positional differences in repeated-sprint ability across conditions. The protocol involved linear sprinting under controlled conditions and did not incorporate multidirectional actions, tactical decision-making, or opponent interactions, which may influence fatigue responses during competitive play. In addition, the condition performed at maximal aerobic speed should be interpreted as a controlled high inter-sprint locomotor load rather than as representative of typical match behavior. Finally, maximum running speed was estimated from the 30–40 m split, which may underestimate true peak instantaneous sprint speed compared with direct measurement; however, this approach is commonly used in field-based protocols. Future research should examine responses under different movement patterns and contextual constraints, as well as explore how varying levels of between-sprint locomotor load influence fatigue development across player profiles.

## 5. Conclusions

In conclusion, this study compared the effects of two between-sprint running intensities, anchored to physiological landmarks (i.e., lactate threshold and maximal aerobic speed), on repeated-sprint performance. The findings indicate that the intensity of running performed between sprint efforts influences sprint performance outcomes under controlled experimental conditions designed to examine responses to different levels of locomotor load. Moderate running below the lactate threshold was associated with greater performance decrements across repeated sprints compared with the passive condition, despite similar blood lactate responses. Higher running intensity at maximal aerobic speed was associated with larger performance decrements and elevated cardiovascular and metabolic responses. Within the present protocol, the passive condition was associated with relatively stable sprint performance during two sets of six repeated 30 m sprints separated by 1 min intervals. These findings highlight the importance of considering between-sprint locomotor load when evaluating repeated-sprint performance, and should be interpreted within the context of a controlled physiological model designed to examine fatigue responses under manipulated between-sprint running intensities.

## Figures and Tables

**Table 1:** Preliminary test results. Values are mean ± SD.

Yo-Yo IR1 distance (m)	1861 ± 226
VO_2_max (mL^−1^·kg^−1^·min^−1^)	52.2 ± 2.1
HRmax (beats/min)	199 ± 4
MAS (km·h^−1^)	16.7 ± 0.4
Max sprinting speed (km·h^−1^)	31.5 ± 1.3
LT1 (km·h^−1^)	10.8 ± 0.8

Yo-Yo IR1 distance (m): distance covered during the Yo-Yo Intermittent Recovery Test Level 1; VO_2_max (mL^−1^·kg^−1^·min^−1^): predicted maximum oxygen consumption from the Yo-Yo IR1; HRmax: peak heart rate achieved during the Yo-Yo IR1; MAS: maximum aerobic speed, running speed attained during the Yo-Yo IR1 test; Max sprinting speed (km·h^−1^): the peak speed achieved in the last 10 m of the 40 m test (between 30 and 40 m); LT1: speed corresponding to the first lactate threshold.

**Table 2:** Sprint performance time for the first part (0–15 m) and the second part (15–30 m) of each 30 m sprint during the three conditions. Values are mean ± SD.

		**First Part of the 30 m Sprint (0–15 m)**										
	Sprint Number (Set 1)						Sprint Number (Set 2)					
**Condition**	1	2	3	4	5	6	1	2	3	4	5	6
Passive (s)	2.44 ± 0.07	2.40 ± 0.08	2.43 ± 0.09	2.45 ± 0.11	2.45 ± 0.07	2.43 ± 0.07	2.45 ± 0.10	2.48 ± 0.11	2.45 ± 0.06	2.45 ± 0.08	2.48 ± 0.08	2.50 ± 0.09
MOD (s)	2.49 ± 0.10	2.45 ± 0.14	2.54 ± 0.12	2.51 ± 0.13	2.52 ± 0.15	2.53 ± 0.10 ^†^	2.50 ± 0.12	2.51 ± 0.09	2.59 ± 0.10 ^†^	2.56 ± 0.12 ^†^	2.62 ± 0.13 ^†^	2.57 ± 0.13 ^†^
HIGH (s)	2.49 ± 0.10	2.55 ± 0.12 ^†^	2.55 ± 0.12 ^†^	2.58 ± 0.12 ^†^	2.59 ± 0.13 ^†^	2.62 ± 0.15 ^†^	2.54 ± 0.11 ^†^	2.59 ± 0.10 ^†^	2.62 ± 0.13 ^†^	2.63 ± 0.15 ^†^	2.70 ± 0.16 ^†#^	2.70 ± 0.17 ^†#^
		**Second Part of the 30 m Sprint (15–30 m)**										
	Sprint Number (Set 1)						Sprint Number (Set 2)					
**Condition**	1	2	3	4	5	6	1	2	3	4	5	6
Passive (s)	1.79 ± 0.08	1.78 ± 0.08	1.77 ± 0.07	1.80 ± 0.05	1.78 ± 0.04	1.78 ± 0.11	1.77 ± 0.06	1.79 ± 0.06	1.78 ± 0.06	1.83 ± 0.07	1.81 ± 0.08	1.82 ± 0.07
MOD (s)	1.78 ± 0.06	1.81 ± 0.07	1.80 ± 0.08	1.82 ± 0.07	1.82 ± 0.08	1.85 ± 0.07 †	1.77 ± 0.07	1.84 ± 0.08	1.83 ± 0.08	1.88 ± 0.08	1.87 ± 0.09	1.88 ± 0.11
HIGH (s)	1.77 ± 0.08	1.83 ± 0.07	1.85 ± 0.09 ^†^	1.87 ± 0.08 ^†^	1.89 ± 0.11 ^†^	1.89 ± 0.10 ^†^	1.79 ± 0.08	1.88 ± 0.07 ^†^	1.91 ± 0.11 ^†#^	1.95 ± 0.11 ^†#^	1.93 ± 0.10 ^†#^	1.95 ± 0.11 ^†#^

MOD: running between sprints at a speed corresponding to 95% of the first lactate threshold (LT1); HIGH: running between sprints at maximal aerobic speed; †: *p* < 0.01 from the corresponding sprint of the passive condition; #: *p* < 0.01 from the corresponding sprint of the MOD condition.

**Table 3:** Mean and peak heart rate responses per condition and sprint set (set 1 and set 2). Values are mean ± SD and are expressed as a percentage of maximal heart rate attained during the Yo-Yo IR1 test.

	Mean Heart Rate		Peak Heart Rate	
Condition	Set 1	Set 2	Set 1	Set 2
Passive	74.0 ± 4.0%	76.6 ± 4.1% *	87.7 ± 2.8%	89.2 ± 2.7% *
MOD	82.1 ± 3.0% †	84.7 ± 3.1% *†	92.7 ± 2.2%	95.1 ± 1.9% *†
HIGH	83.2 ± 3.8% †	86.3 ± 4.0% *†	94.9 ± 1.4%	95.7 ± 1.9% *†

MOD: running between sprints at 95% of lactate threshold; HIGH: running between sprints at maximum aerobic speed—MAS; *: *p* < 0.01 from set 1; †: *p* < 0.01 from passive.

## Data Availability

The data presented in this study are openly available in [Figshare.com] at [10.6084/m9.figshare.30953789].

## Abbreviations

The following abbreviations are used in this manuscript:

MAS (km·h^−1^)	Maximal aerobic running speed
MOD	Running between sprints at a speed corresponding to 95% of the first lactate threshold
HIGH	Running between sprints at a speed corresponding to the maximum aerobic speed
Yo-Yo IRT Level 1	Yo-Yo Intermittent Recovery Level 1 Test
VO_2_max	Maximum oxygen uptake relative to body mass (mL^−1^·kg^−1^·min^−1^)
LT_1_	Speed corresponding to the first lactate threshold
PCr	Phosphocreatine
